# Hypothermia Increases Tissue Plasminogen Activator Expression and Decreases Post-Operative Intra-Abdominal Adhesion

**DOI:** 10.1371/journal.pone.0160627

**Published:** 2016-09-01

**Authors:** Meng-Tse Gabriel Lee, Chien-Chang Lee, Hsuan-Mao Wang, Tzung-Hsin Chou, Meng-Che Wu, Kuang-Lung Hsueh, Shyr-Chyr Chen

**Affiliations:** 1 Department of Emergency Medicine, National Taiwan University Hospital and National Taiwan University College of Medicine, Taipei, Taiwan; 2 Department of Emergency Medicine, National Taiwan University Hospital Yunlin Branch, Douliou, Taiwan; 3 Department of General Medicine, National Taiwan University Hospital Yunlin Branch, Douliou, Taiwan; 4 Research Department, China Medical University Hospital, Research Center for Tumor Medical Science, and Center for Chinese medicine and acupuncture, China Medical University, Taichung, Taiwan; Max Delbruck Centrum fur Molekulare Medizin Berlin Buch, GERMANY

## Abstract

**Background:**

Therapeutic hypothermia during operation decreases postoperative intra-abdominal adhesion formation. We sought to determine the most appropriate duration of hypothermia, and whether hypothermia affects the expression of tissue plasminogen activator (tPA).

**Methods:**

80 male BALB/c mice weighing 25–30 g are randomized into one of five groups: adhesion model with infusion of 15°C saline for 15 minutes (A); 30 minutes (B); 45 minute (C); adhesion model without infusion of cold saline (D); and sham operation without infusion of cold saline (E). Adhesion scores and tPA levels in the peritoneum fluid levels were analyzed on postoperative days 1, 7, and 14.

**Results:**

On day 14, the cold saline infusion groups (A, B, and C) had lower adhesion scores than the without infusion of cold saline group (D). However, only group B (cold saline infusion for 30 minutes) had a significantly lower adhesion scores than group D. Also, group B was found to have 3.4 fold, 2.3 fold, and 2.2 fold higher levels of tPA than group D on days 1, 7, and 14 respectively.

**Conclusions:**

Our results suggest that cold saline infusion for 30 minutes was the optimum duration to decrease postoperative intra-abdominal adhesion formation. The decrease in the adhesion formations could be partly due to an increase in the level of tPA.

## Introduction

Postoperative intra-abdominal adhesion after laparotomy is a source of considerable morbidity. Up to 70% of patients who had prior laparotomies develop intra-abdominal adhesion [1–3]. Postoperative adhesions affect the quality of life in millions of people worldwide, causing many different types of complications, including chronic pelvic or abdominal pain, small bowel obstructions, and even infertility[4, 5]. Small bowel obstruction is the most common complication of adhesion and is observed in up to 70% of patients undergoing laparotomy[6–8]. Although less commonly observed, up to 20% of female infertility has been associated with postoperative adhesions[9, 10].

The pathogenesis of postoperative intra-abdominal adhesion is not completely understood, but is thought to involve a complex interplay between clot formation, fibrinolysis, and wound healing [11–13]. Peritoneal fibrinolytic activity has been shown to play a key role in the pathophysiology of adhesion [14, 15]. Tissue plasminogen activator (tPA), is an enzyme that can catalyze the degradation of fibrin gel matrix into fibrin split products. Fibrin split products have less effect on adhesion formation. The activity of tPA is in turn controlled by plasminogen activator inhibitor-1 (PAI-1) [16].

Several clinical and animal studies have demonstrated a strong correlation between adhesion formation and tPA activity. Also, after injury or surgery, the activity and concentration of tPA decrease [17–19]. However, the direct manipulation of the tPA level (to reduce the adhesion formation) can only be conducted through the addition of recombinant tPA. Several researchers demonstrated that recombinant tPA can reduce adhesion formation by up to 90% in different animal models [19–25]. Unfortunately, one group found that there are hemorrhagic complications with use of recombinant tPA [26]. Thus, more pharmacokinetics and safety study are suggested for the clinical use of recombinant tPA in the prevention of post-operative adhesion [25].

Several groups, including ours, have shown that therapeutic hypothermia during operation decreases postoperative intra-abdominal adhesion formation [27–30]. Previously, we have determined that 15°C saline infusion is the optimum temperature for decreasing the adhesion formation, but the optimum duration for therapeutic hypothermia was uncertain [29]. Therefore, the first aim of this study is to determine the optimum duration for hypothermia. Secondly, we wished to investigate whether hypothermia can cause a decrease in postoperative intra-abdominal adhesion formation via changes in expression of tPA and PAI-1. This is because tPA and PAI-1 have been shown to be important in adhesion formation, and hypothermia has been found to affect the expression of enzymes [31, 32].

## Methods

### Ethical approval

This study was approved by the institutional review board of National Taiwan University Hospital.

### Experimental Animals

The protocols that are used in this study have been approved by the National Taiwan University Hospital Animal Protocol Review Committee (approval ID: 20120285). All the protocols are in adherence to the guidelines established in the Guide for the Care and Use of Laboratory Animals of the National Health Research Institutes. BALB/c mice were obtained from BioLasco Taiwan, a technology licensee of Charles River Laboratories in Taiwan. The mice were kept on a standard 10-hour light and 14-hour dark cycle and allowed free access to water and food.

To alleviate pain and suffering, the mice were anesthetized with intraperitoneal pentobarbital (50 mg/kg of body weight) before the operation. Using a previously described procedure, a low 2-cm midline laparotomy was performed for five different groups of mice [29]. 80 male BALB/c mice weighing 25–30 g are randomized into one of the five groups: (A) adhesion model with infusion of 15°C normal saline for 15 mins (N = 18); (B) adhesion model with infusion of 15°C normal saline for 30 mins (N = 18); (C) adhesion model with infusion of 15°C normal saline for 45 mins (N = 18); (D) adhesion model without infusion of cold saline and the abdominal cavity was left open for 15 mins (N = 17); (E) sham operation without infusion of cold saline and the abdominal cavity was left open for 15 mins (N = 9). After the predefined length of cold saline infusion or opening of the abdominal cavity, we closed the abdominal incision with absorbable surgical sutures.

We used a slightly modified standard adhesion model [33]. Briefly, the anterior cecal wall was abraded for 20 strokes with dry sterile gauze, and full-thickness 4–0 silk sutures were also placed in the traumatized anterior cecal wall to increase adhesion formation. Localized hypothermia was induced by continuous infusion of cold saline through a miller catheter placed at the cephalic part of the abdominal cavity. The excess cold saline was drained out through another Miller catheter located in the caudal part of the abdominal cavity. Since the goal of the experiment is to decrease adhesion formation in the peritoneal cavity and not to cause whole body hypothermia, we only determined the change in the abdominal cavity temperature by inserting an electrical temperature probe into the abdominal cavity before and after the normal saline infusion

### Adhesion score interpretation

On the 1, 7, or 14 days after surgery, mice were sacrificed by cervical dislocation. A well-documented adhesion-severity scoring system is used by the Adhesion Scoring Group to measure the degree of adhesion formation [34]. The Adhesion Scoring Group based their adhesion scoring system on the evaluation of 23 individual locations in the abdominal cavity for severity (S) (0: none; 1: filmy, avascular; 2: dense and/or vascular; 3: cohesive), and extent of total area or length (A) (0: none; 1: ≤ 25%; 2: 26–50%; 3: > 50%). We sum up the individual S and A value to be (S + A) for each mouse to present the adhesion condition from 0 to 6, from least to most adhesion intensity.

### tPA and PAI-1

Peritoneal fluid was removed after the operation by infusing abdominal cavity with normal saline. The peritoneal fluid was centrifuged at 9000g for 15min at 4°C, before freezing the supernatant at -80°C. Aliquots of the thawed supernatants were analyzed for total antigen concentration of tPA and PAI-I, using commercially available ELISA kits from Molecular Innovations (Novi, USA). Total protein content was determined by Bradford assay (Sigma, USA).

### Data Analysis

All the values presented are mean ± standard error. Due to the relatively small sample size, we cannot make any assumption to the distribution of the variable. Thus, means are compared between the different groups of mice using the Mann-Whitney *U* test. A P value of <0.05 was considered statistically significant. The statistical analyses were carried out using SPSS statistical package (version 21.0, IBM SPSS Statistics, IBM Corporation, Armonk, NY).

## Results

### Temperature

The mean (±SE) peritoneal cavity temperature for the adhesion model mice (group D) and the sham mice (group E) were determined 15 minutes after performing midline laparotomy. We found the mean peritoneal temperature for group D and E decreases to a similar extent (31.11 ± 0.30 and 31.56 ± 0.33°C, respectively). The peritoneal cavity temperature for the groups of mice undergoing hypothermia (group A, B, C) was determined after fitting with Miller catheter, but before cold saline infusion. After fitting with Miller catheter, mice that would be undergoing hypothermia were observed to have a lower mean peritoneal temperature than group D and E (Group A: 30.08 ± 0.33; B: 30.35 ± 0.21; C: 30.88 ± 0.22°C). After different duration of cold saline infusion, there was approximately 6–7°C drop in the abdominal cavity temperature for the mice undergoing hypothermia. As expected, longer duration of cold saline infusion led to a lower mean abdominal temperature (Group A: 23.98 ± 0.41; B: 23.54 ± 0.49; C: 23.12 ± 0.39°C).

### Intra-abdominal adhesion score

**Fig 1:**
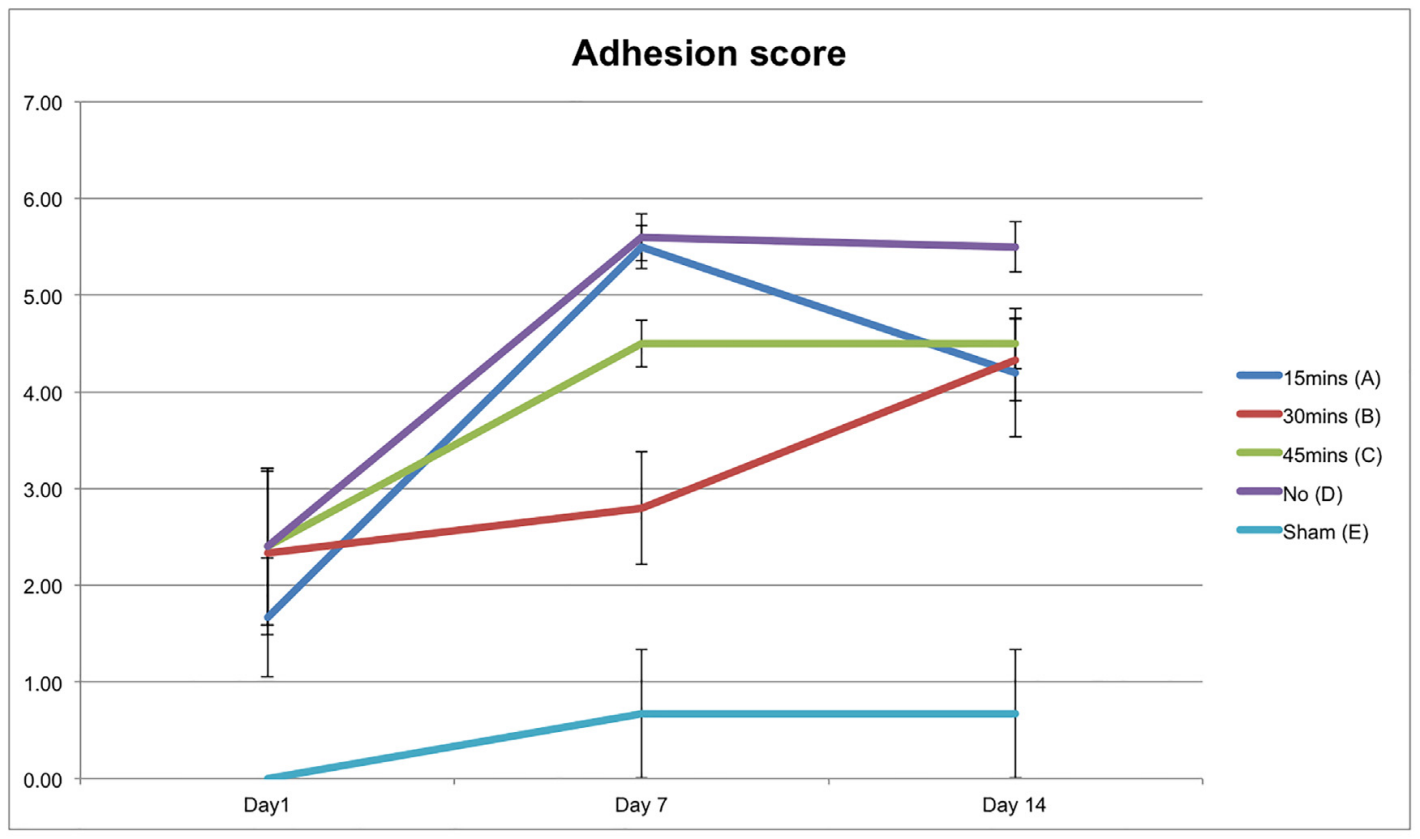
Intra-abdominal adhesion score.

The mean (±SE) adhesion scores were plotted for postoperative day 1, 7, and 14 (Fig 1). In general, there are little adhesion formations on day 1, and the adhesion score increases over time. We observed that the sham group (group E in cyan), which had not undergone the adhesion model, had the lowest adhesion score as expected. Group D (in purple, the mice that have undergone adhesion model but not hypothermia) had the highest adhesion score throughout the study period. On day 14, all the groups (group A, B, and C) that have undergone cold saline infusion were associated with lower adhesion scores than group D. However, only group B (30 minutes duration) has significantly lower adhesion scores than group D on day 14 (p<0.05).

### tPA level in the peritoneum fluid

The mean (±SE) tPA levels in the peritoneum fluid were reported in Table 1. In general, tPA level increased from day 1 to day 7 for all groups, but decreased again to the baseline levels on day 14. We observed that the sham group of mice had the lowest mean tPA level throughout the study period. Group D (undergone adhesion model but not hypothermia) generally had comparable tPA level to the sham group. However, group D had lower tPA level than the groups that had hypothermia (group A, B, and C). The difference between group B and group D is significant on day 1 (p <0.05) and nearly significant on day 7 and day 14 (p < 0.1). The difference between group C and group D is significant on day 1 (p <0.05) and nearly significant on day 7 (p < 0.1).

**Table 1:** tPA level in the peritoneum fluid.

Group\ Time	Day 1 (ng/ml)	Day 7 (ng/ml)	Day 14 (ng/ml)
A (15 mins)	0.154 ± 0.122	0.334 ± 0.188 *	0.040 ± 0.007
B (30 mins)	0.299 ± 0.078 **	0.438 ± 0.095 *	0.244 ± 0.049 *
C (45 mins)	0.316 ± 0.057 ***	0.445 ± 0.168 *	0.409 ± 0.248
D (No)	0.088 ± 0.027	0.190 ± 0.162	0.111 ± 0.049
E (Sham)	0.057 ± 0.017	0.173 ± 0.053	0.043 ± 0.003
B/ D	3.41	2.31	2.19
C/ D	3.61	2.34	3.67

* refers to p<0.1,

** refers to p<0.05 D,

*** refers to p<0.01.

All the comparison for the group of interest is made towards group D on the same day.

### PAI-I level in the peritoneum fluid

The mean (±SE) PAI-I levels in the peritoneum fluid were shown in Table 2. In general, the PAI-I levels are similar between group A, D, and E on the different post-operative days. The only exception is on day 1, in which group D, the animals that have not undergone hypothermia were observed to have significantly higher concentration of PAI-I than group A, B, C and E. On post-operative day 1, group D was found to have 2.3 fold, 5.2 fold, and 15.4 fold higher respective levels of PAI-I than group A, B, and C; the results are all significant (p < 0.05). Group D also had a higher level of PAI-I than the sham group (E) on post-operative day 1, but the result is only nearly significant (p < 0.1). On post-operative days 7 and 14, however, group D no longer had a significantly higher level of PAI-I than the groups that had undergone hypothermia (group A, B, and C). Instead, group D was found to have significantly lower level of PAI-I than group B on post-operative day 7 and day 14.

**Table 2:** PAI-I level in the peritoneum fluid.

Group\ Time	Day 1 (ng/ml)	Day 7 (ng/ml)	Day 14 (ng/ml)
A (15mins)	8.354 ± 2.164 ***	2.539 ± 0.998	0.518 0078 0.161
B (30mins)	3.630 ± 0.776 ***	9.428 ± 1.204 **	3.643 ± 1.166 **
C (45mins)	1.221 ± 0.446 ***	5.156 ± 1.175	0.993 ± 0.381
D (No)	18.858 ± 8.694	2.930 ± 0.483	0.814 ± 0.158
E (Sham)	8.033 ± 2.801*	3.933 ± 4.126	0.582 ± 0.104
D/B	5.20	0.31	0.22
D/C	15.44	0.57	0.82

* refers to p<0.1,

** refers to p<0.05 D,

*** refers to p<0.01.

All the comparison for the group of interest is made towards group D on the same day.

## Discussion

Several groups have reported that therapeutic hypothermia during operation could decrease postoperative intra-abdominal adhesion formation. However, the most appropriate duration of hypothermia and the working mechanism on how hypothermia could decrease postoperative intra-abdominal adhesion formation is unclear. This study found that all the groups of mice that have undergone hypothermia were associated with lower adhesion scores than the mice without hypothermia on post-operative day 14. However, only the group of mice that had undergone hypothermia for 30 minutes had significantly lower adhesion scores than the group of mice that had not undergone hypothermia. Correspondingly, we found that the 30 minutes hypothermia group had 3.4 fold (day 1), 2.3 fold (day 7), and 2.2 fold (day 14) higher level of tPA than the group without hypothermia.

A few clinical studies have demonstrated that there is a reduction in the level of peritoneal tPA after injury. Brokelman *et al*. reported that patients undergoing laparoscopic gastric bypass surgery showed a 30% decrease in tPA concentration after 90 minutes of surgery [17]. Scott-Coombes *et al*. carried out a longer study and reported that in patients undergoing elective laparotomy for non-inflammatory disease; peritoneal tPA level decreased by more than 2 fold on post-operative day 1 [19]. Thus, researchers tried to use recombinant tPA to reduce adhesion formation after recombinant tPA became commercially available. Lai *et al*. found that adding 30 ug of recombinant tPA to Wister rats immediately after the operation can decrease post-operative adhesion on day 14 [23]. Evans *et al*. found that adding 800 ug of recombinant tPA to Sprague-Dawley rats for 4 continuous days can decrease ischemic button adhesion on day 7 [24]. Compared to the group of mice that had undergone adhesion but not hypothermia, our study found that the tPA level for the group of mice that had undergone 30 minutes hypothermia showed an increase of approximately 0.21 ng/ml of tPA on post-operative day 1 and an increase of approximately 0.13 ng/ml of tPA on post-operative day 14. It is difficult to determine the exact increase of the tPA level for each group of mice, but we can estimate a consistent increase in tPA level in the low ng range for the group of mice that have undergone 30 minutes of hypothermia.

Due to differences in the study design, our results cannot be directly compared to the previous reports using recombinant tPA. However, it is clear from comparison with previous reports on recombinant tPA that the rise in the concentration of tPA due to hypothermia may not be the sole reason for the observed decrease in post-operation adhesion. In fact, hypothermia has been shown to influence multiple processes such as cellular metabolism, expression of metalloproteinase and inflammation [31, 32, 35]. The mechanism on how hypothermia can reduce inflammation has been well characterized. Hypothermia has been found to decrease inflammation by decreasing both the infiltration of polymorphonuclear cells and the production of a wide range of inflammatory proteins such as TNF-α, IL-1b, IL-6, and macrophage inflammatory protein-2. [29, 36, 37]

Currently, the most well-characterized approach to prevent post-operative adhesion is to use a biodegradable barrier, which can be either solid or fluid based. Meta-analysis of 28 randomized controlled trials (5191 patients) suggested that the two commercially available solid barriers: oxidized regenerated cellulose or carboxymethylcellulose can effectively and safely prevent post-operative adhesion [38]. However, during laparoscopic surgery, solid barriers have the disadvantage owing to the difficulty to apply. Icodextrin and polyethylene glycol are two fluid-based barriers that have the theoretical advantage of easier administration and cover more potential sites of adhesion formation. However, the same meta-analysis did not show that either icodextrin or polyethylene to significantly reduce post-operative adhesion [38].

In the future, it will be interesting to test the clinical application of hypothermia in laparotomy and see how well it compares with currently available biodegradable barriers. In our study, we use a standard Miller catheter for the induction of hypothermia, which has the advantage of localized cooling at the site of operation, and relatively rapid cooling rates. In humans, the peritoneal cavity is much larger, and it is conceivable to use the Celsius Control system (Innercool Therapies, Inc) [39]. The Celsius Control system is an intravascular cooling catheter system that been designed and approved for clinical use in neurosurgical patients. Use of therapeutic hypothermia in brain injury patients has been well characterized, and moderate hypothermia has been shown to improve the clinical outcome of these patients.[40, 41] Unfortunately, to the best of our knowledge, there is still no clinical use of therapeutic hypothermia in laparotomy. Given the proven clinical benefit of hypothermia in brain injury patients and our results showing that hypothermia decreases post-operative adhesion, it might be worthwhile for clinicians to test therapeutic hypothermia on patients undergoing laparotomy next.

Our study has some inherent limitations that need to be clarified in future studies. First, our adhesion scoring system only investigates cecal adhesions and did not address the tenacity and extent of adhesions to a patch. It will be noteworthy to find out whether using a silicone patch model will give rise to a different result. Second, it will be interesting to find out whether other factors of the fibrinolysis system are also affected by hypothermia. Third, the mechanism on why 45 minutes of hypothermia result in a worse adhesion score than 30 minutes is currently unclear. Postoperative intra-abdominal adhesion complex is attributed to a complex interplay between clot formation, fibrinolysis, and wound healing. In this study, we found that 30–45 minutes of hypothermia is likely to increase fibrinolysis due to increase in expression of tPA. However, it is still unclear how will 30–45 minutes of hypothermia affects clot formation. Since the coagulation cascade starts to be affected when the whole body temperature drops to 34°C, it is likely that hypothermia for 45 minutes affects the coagulation cascade to a greater extent than hypothermia for 30 minutes [42]. In the future, it will be interesting to systemically investigate the complex association between whole body temperture, peritoneal cavity temperature, coagulation and post-operative adhesion.

To conclude, our results suggest that cold saline infusion for 30 minutes was the most optimum duration to decrease postoperative intra-abdominal adhesion formation. Our results also suggest that one mechanism that cold saline infusion can decrease postoperative intra-abdominal adhesion formation is through an increase in the concentration of tPA.
